# Reproductive outcomes in fresh transfer cycles and antagonists with premature luteinizing and/or progesterone surge: a single center retrospective cohort study

**DOI:** 10.3389/fendo.2024.1411106

**Published:** 2024-09-24

**Authors:** Chun-Xiao Wei, Jian-Wei Zhang, Shan Xiang, Fang Lian

**Affiliations:** ^1^ First Clinical Medical College of Shandong University of Traditional Chinese Medicine, Jinan, Shandong, China; ^2^ Shandong University of Traditional Chinese Medicine Affiliated Hospital, Jinan, Shandong, China

**Keywords:** luteinizing hormone surge, progesterone, flexible antagonist protocol, fresh cycle, live birth rate

## Abstract

**Background:**

The optimal outcome of assisted reproductive technology is a successful live birth after fresh embryo transfer. However, the success pregnancy rate of fresh embryo transfer cycle in antagonist protocol is lower than that observed in other protocols. Despite the use of antagonists (GnRH-ant), the incidence of luteinizing hormone surge and elevated progesterone levels remain at approximately 5%-38%. Progesterone is widely recognized to exert adverse effects on fresh embryo transfer outcomes. This study aimed to investigate the impact of luteinizing hormone surge and progesterone levels on live birth rate following fresh embryo transfer and explore appropriate progesterone thresholds to enhance pregnancy outcomes.

**Methods:**

This retrospective cohort study included a total of 1,177 antagonist protocol cycles with fresh embryo transfer. The patients were divided into four groups based on the presence of premature LH surge and progesterone level on trigger day>1.5ng/ml. Then, the relationship between the variables and the pregnancy outcome was analyzed and compared in each group.

**Results:**

The transient rise of luteinizing hormone did not impact pregnancy outcomes (P=0.345; P=0.3; P=0.787), in contrast to progesterone levels on the day of hCG administration (P=0.047*; P=0.015*; P=0.021*). In cases with luteinizing hormone surge, elevated progesterone levels were correlated with higher antral follicle count (AFC), and as progesterone levels increased, a greater quantity of oocytes and embryos were obtained. However, there was no statistically significant difference in pregnancy outcomes. In cases without luteinizing hormone surge, elevated progesterone levels led to significantly poorer pregnancy outcomes. Furthermore, the curve-fitting and threshold-effect analysis revealed a notable decline in live birth rates when progesterone exceeded or equaled 1.10ng/ml (OR, 0.25; 95% CI, 0.09–0.66; P = 0.005*).

**Conclusion:**

The GnRH-ant dosage addition should be carefully selected in flexible antagonist protocols. The presence of elevated progesterone levels may be associated with improved embryo quality when luteinizing hormone surge occurred. In the absence of a luteinizing hormone surge, progesterone levels showed a larger impact on the pregnancy outcome, and fresh embryo transfer should not be performed if the progesterone level on the day of hCG administration is higher than 1.10ng/ml.

## Introduction

During *in vitro* fertilization (IVF) and intracytoplasmic sperm injection (ICSI) cycles, controlled ovarian hyperstimulation (COH) plays an essential role. The GnRH antagonist protocol employs exogenous gonadotropin (Gn) to stimulate follicle development, which competitively occupies the pituitary GnRH receptor using a gonadotropin-releasing hormone antagonist (GnRH-ant); the internal luteinizing hormone (LH) peak is thus controlled and early ovulation or luteinization of follicles is effectively inhibited. In comparison to the GnRH agonist protocol, the GnRH-ant protocol allows for timely adjustments based on follicle development. It offers enhanced efficiency, convenience, and safety by avoiding premature pituitary suppression and reducing the risk of ovarian hyper-stimulation syndrome (OHSS) (1). Over the past two decades, GnRH antagonists have replaced GnRH agonists as the preferred treatment for ART (2).

Fresh embryo transfer (ET) can reduce the time and economic burden on patients, as compared to frozen embryo transfer (FET). Patients undergoing frozen embryo transfer require additional medical care and monitoring, endometrial preparation, embryo freezing, and thawing procedures. Moreover, studies have suggested that frozen embryo transfer increases the risk of adverse pregnancy outcomes such as placenta praevia, preeclampsia, and an increased risk of leukemia due to the aforementioned procedures involved in freezing and thawing embryos (3–6). The desired outcome in assisted reproductive technology (ART) is a successful live birth through fresh embryo transfer. However, the pregnancy rate of fresh embryo transfers in patients receiving the GnRH-ant protocol is consistently lower than other protocols (7). Additionally, patients undergoing the GnRH-ant protocol are more susceptible to premature luteinizing hormone surge and elevated progesterone levels (8–11). The LH surge predominantly manifests in individuals with low and high ovarian responsiveness (12–15).

Researchers reported that LH level changes were associated with the outcome of COH. The peak LH level may be used to predict optimal oocyte yield, providing superior predictive accuracy than basal or trigger-day LH levels (16). The peak/basal LH ratio may potentially be used to predict the pregnancy outcomes in PCOS patients, and patients with a peak/basal LH ratio≥ 1 exhibiting a higher top-quality embryo (17). However, other scholars suggested that a transient premature LH surge without elevated serum progesterone may cause a detrimental effect on the embryo and pregnancy outcomes in fresh embryo transfer cycles (14, 18, 19) and on the ongoing pregnancy rate (1, 20), especially the patients of advanced age (≥37 years), and aggravated the reduced potential of embryos growth, not the number (21). However, previous studies explored patients who experienced a transient premature rise in LH and had comparable clinical pregnancy and ongoing pregnancy rates (22–24). The current research on the relationship between the LH surge and pregnancy outcomes remains controversial, but the LH surge is widely recognized to result in an elevation of P levels due to premature luteinization of granulosa cells or ovulation of small follicles. The adverse effects of elevated P levels on the trigger day have been extensively documented. These effects encompass not only impaired endometrial receptivity but also deleterious consequences on embryo quality (3, 25–28). Xu B et al. proposed that the ongoing pregnancy rate in fresh cycles was negatively correlated with serum P levels on the day of hCG administration, and different threshold concentrations of P were determined based on varying ovarian responses (29, 30). The literature suggested that patients undergoing fresh blastocyst transfer should aim for a P level of 0.8 ng/ml on the day of hCG administration, in contrast to fresh embryo transfer (31). However, the progesterone level threshold affecting the level of LH and pregnancy outcomes has not been clearly established.

In clinical practice, appropriate LH levels and progesterone levels are critical in ensuring the acquisition of mature eggs and a satisfactory pregnancy outcome. This study aimed to identify the reasons underlying the low success rate of fresh embryo transfer in the antagonist protocol and to improve the clinical pregnancy rate of fresh embryo transfer.

## Materials and methods

### Population and study subjects

This retrospective analysis included 1,177 infertile patients who underwent IVF/ICSI cycles and fresh embryo transfer from May 2017 to December 2022. The women were monitored until live birth after fresh embryo transfer. The study protocol was approved by the institutional human ethics committee.

The inclusion criteria were: (1) flexible antagonist protocol with fresh embryo transfer cycle, (2) basal follicle-stimulating hormone (bFSH) ≤ 15 IU/L, and (3) body mass index (BMI) <40kg/m^2^.

The exclusion criteria were: (1) patients with chromosomal abnormalities, reproductive malformation, and a history of recurrent spontaneous abortion; (2) patients undergoing coasting to prevent ovarian hyperstimulation syndrome; (3) incomplete information; (4) uterine pathologies that might compromise pregnancy potential; (5) patients with elevated progesterone or LH level before the addition of GnRH-ant.

The premature LH surge refers to an endogenous LH peak that occurs before follicle maturation or human chorionic gonadotrophin (hCG) injection. It was defined as either maximum LH levels exceeding two and a half times the baseline LH level on day 2 of the same menstrual cycle (13), or the absolute value > 10 IU/mL (32–34).

#### MLH grouping

The populations were categorized into two groups based on the presence of a transient premature maximum luteinizing hormone surge. Group MLH0 was defined as the group without transient premature luteinizing hormone surge, whereas group MLH1 included patients with transient premature luteinizing hormone surge.

#### P grouping

The populations were categorized into two groups based on the P level on hCG day. Patients with P ≤ 1.5 ng/ml were assigned to group P0, and those with P>1.5 ng/ml were assigned to group P1.

#### PMLH grouping

Furthermore, the patients were divided into four groups according to the P level on hCG day and the presence of premature LH surges. Group P1MLH1 included patients with transient premature luteinizing hormone surges and P>1.5 ng/ml; group P1MLH0 included those without transient premature luteinizing hormone surges and P>1.5 ng/ml; group P0MLH1 comprised patients with transient premature luteinizing hormone surges and P ≤ 1.5 ng/ml; group P0MLH0 consisted of individuals without transient premature luteinizing hormone surges and P ≤ 1.5 ng/ml.

### Ovarian stimulation protocol

Patients received IVF/ICSI treatment according to the flexible GnRH antagonist protocol. On the second day of the menstrual cycle, recombinant human follicle-stimulating hormone 150–300 U (Gonal-F; Merck, Lyon, France; Puregon, MSD, Boulogne, France) was injected as Gn. The Gn doses were determined based on patient age, body mass index (BMI), FSH, and AFC. A daily dose of 0.25/0.125 mg of GnRH-ant (Cetrotide, Merck, Lyon, France) was administered when the leading follicle size was 14 mm or the serum E2 level reached 300pg/mL or a premature luteinizing hormone surge was recognized.

### Oocyte retrieval and transfer

Oocytes were then collected by follicular aspiration under ultrasound 34–36 h after triggering with GnRH-a (Triptoreline, Decapeptyl, Ipsen, France) or recombinant hCG (rhCG, Ovitrelle, Merck, Lyon, France). IVF or ICSI fertilization was selected according to semen conditions. Eighteen hours after fertilization, embryo development was monitored daily and graded based on the number and size of blastomeres, fragmentation rate, multinucleation, and early densification. High-quality embryos were defined as embryos with seven to ten blastomeres on the third day following oocyte retrieval (35).

All patients completed an IVF/ICSI cycle and then performed an ET cycle. On day 3 or day 5, one or two of the best embryos or blastocysts were selected and transferred using a soft Wallace catheter. In order to avoid multiple pregnancies and reduce the incidence of complications during pregnancy and the perinatal period, the number of embryos was selected according to the specific conditions of the patient and the embryo quality score, and one blastocyst was selected as far as possible. For luteal support in preparation for the fresh embryo transfer, 40mg of progesterone (20 mg/branch, Zhejiang Xianju Pharmaceutical Co., Ltd.) was injected daily and 30mg of oral dydrogesterone tablets (10 mg/tablet, Abbott Healthcare Products B.V.) or progesterone vaginal sustained-release gel (90 mg/dose, Crinone VR 8%, Merck, Sherano, Switzerland) were given daily. In addition, two bags of Chinese medicine, the Gushen Antai pills, were used daily.

### Hormone measurement

Venous blood samples were collected on the second day of the menstrual cycle to the triggering day. Serum FSH, LH, E_2,_ and P levels were measured by means of the automated Elecsys Immunoanalyzer (Beckmann, America).

### Pregnancy outcomes measurement

The live birth rate (LBR) was the primary outcome. The secondary outcomes included biochemical pregnancy rate (BPR), clinical pregnancy rate (CPR), number of oocytes, number of fertilization, number of blastomeres, number of embryos, and number of high-quality embryos/blastomeres.

Live birth was defined as live birth per embryo transfer in a fresh cycle.

Clinical pregnancy was defined as the presence of an intrauterine gestational sac with a fetal heartbeat detected on transvaginal ultrasonography after six weeks of gestation.

Biochemical pregnancy was defined as the presence of detectable ß-hCG in the blood and the value<10mIU/mL at approximately 14 days after transplantation.

LBR = number of cycles with live birth/number of transfer cycles × 100%

BPR = number of cycles with biochemical pregnancy/number of transfer cycles × 100%

CPR = number of cycles with clinical pregnancy/number of transfer cycles × 100%

### Statistical analysis

Statistical analysis was conducted using SPSS version 26 and R4.2.1. In this study, P<0.05 was considered statistically significant. The Shapiro–Wilk test was used to assess data normality. Due to skewed distributions, quantitative variables were expressed as medians (interquartile range, range between the 25th and 75th percentiles), and the Mann–Whitney U test and Kruskal-Wallis test were performed. Qualitative variables were expressed as frequencies and analyzed using the chi-square test. Moreover, correlation analysis was performed using Spearman based on the R language. Binary logistic regression analysis was performed based on the following patient characteristics: female age, BMI, AFC, Gn dosage, GnRH-ant dosage, endometrial thickness, number of embryo transfers, high-quality embryos, blastocysts, and group PMLH. The crude odds ratios (OR) and adjusted OR were calculated with 95% confidence intervals (CI). In addition, curve fitting and threshold effect analysis were performed on group MLH0. Smooth curve fitting was carried out with the gam model to identify any non-linear relationship between the P levels and LBR. A piece-wise linear regression method was used to analyze the threshold effect between the P levels and LBR.

## Results

### Baseline characteristics

In total, 1177 patients who received fresh embryo transfer with antagonist regimens from May 2017 to December 2022 were retrospectively analyzed. As displayed in **Table 1**, there were 27 cases in group P1MLH1, 76 cases in group P1MLH0, 135 cases in group P0MLH1, and 939 cases in group P0MLH0. As illustrated in **Table 1**, the age of participants in group P0MLH1 (35(31,39)) was significantly higher than group P0MLH0 (34(31,38), P=0.049*). The BMI of P0MLH0 (23.4(21.3,26.7)) was significantly higher than group P0MLH1 (23(20.75,25.3), P=0.048*). In addition, the AFC of group P1MLH1 24(18.5,28) was significantly higher than group P0MLH1 (16(8,24), P=0.001*) and group P0MLH0 (17(12,24), P=0.011*). The GnRH-ant dosage of group P1MLH0 (1(0.625,1.25)) was the highest (P=0.049*). The baseline FSH of group P0MLH1 (7.55(6.36,9.22)) was the highest (P=0.025*). The baseline LH of group P1MLH1 (5.01(3.38,6.15)) was the highest (P=0.025*). Furthermore, group P1MLH1 showed a significantly higher number of oocytes (11(8,15.5)) than group P0MLH1 (7(3,10.5), P=0.000*) and group P0MLH0 (8(5,11), P=0.007*). Oocyte numbers of group P1MLH0 (9(6,12.5)) was also higher than group P0MLH1 (7(3,10.5), P=0.001*) and group P0MLH0 (8(5,11), P=0.044*). The fertilization numbers of P1MLH1 (6(3.5,9), P=0.025*) and group P1MLH0 (5(4,8), P=0.004*) were higher than group P0MLH1 (4(2,7)). Embryo numbers of group P1MLH1(4(2,6)) were higher than group P0MLH1 (2(1,4), P=0.026*). The blastocyst number of group P1MLH1 (0(0,2)) was the highest (P=0.003*).

**Table 1:** Baseline characteristics between group PMLH.

Parameter	Group P1MLH1	Group P1MLH0	Group P0MLH1	Group P0MLH0	P-Value
Number of cycles	27	76	135	939	
Age, years	33 (28.5, 36.5)	33.5 (31, 37.5)	35 (31, 39)	34 (31, 38)	0.046*
BMI, kg/m^2^	22.8 (21.6, 25.8)	23.4 (21.05,25.7)	23 (20.75, 25.3)	23.4 (21.3,26.7)	0.045*
AFC	24 (18.5,28)	18.5 (14,24)	16 (8,24)	17 (12,24)	0.001*
Gn days	10 (8.5, 11)	9 (8, 10)	9 (8, 10)	9 (8, 10)	0.338
Gn dosage, IU	2360 (1600, 2860)	2137 (1800,2700)	2025 (1575, 2625)	2025 (1700,2600)	0.511
GnRH-ant days	4 (3, 5)	5 (4, 6)	4 (3, 5)	4 (3, 5)	0.071
GnRH-ant dosage, mg	1 (0.75, 1.25)	1 (0.625, 1.25)	0.75 (0.5, 1.25)	1 (0.5, 1.25)	0.049*
Duration of Infertility, years	3 (1, 4)	3 (2, 4)	3 (2, 5)	3 (2, 4)	0.413
Baseline FSH, IU/L	6.80 (5.76, 7.90)	7.71 (6.17, 8.81)	7.55 (6.36, 9.22)	7.30 (6.12, 8.54)	0.025*
Baseline LH, IU/L	5.01 (3.38, 6.15)	4.59 (3.62, 5.68)	4.62 (3.30, 6.57)	4.05 (3.07, 5.30)	0.000*
Baseline E_2_, pg/mL	32.33 (29.25, 47.56)	39.50 (31.22, 52.28)	40.09 (29.85, 49.00)	36.00 (27.49, 48.00)	0.103
Baseline P, ng/mL	0.425 (0.225, 0.54)	0.515 (0.255, 0.625)	0.45 (0.28, 0.58)	0.40 (0.25, 0.63)	0.564
LH (HCG), IU/L	3.43 (1.55, 6.18)	2.01 (1.07, 3.03)	3.42 (2.62, 6.22)	2.28 (1.35, 3.43)	0.000*
E_2_ (HCG), pg/mL	2851 (2059, 3257)	2011 (1680, 3298)	1481 (715, 2078)	1632 (1107, 2295)	0.000*
P (HCG), ng/mL	1.73 (1.63, 1.87)	1.65 (1.60, 1.82)	0.90 (0.61, 1.20)	0.92 (0.62, 1.15)	0.000*
Endometrial thickness, mm	10.0 (8.5, 11.3)	10.0 (10.0, 11.7)	10.0 (9.4, 11.0)	10.0 (9.4, 12.0)	0.470
Number of oocytes	11 (8, 15.5)	9 (6, 12.5)	7 (3, 10.5)	8 (5, 11)	0.000*
Number of fertilization	6 (3.5, 9)	5 (4, 8)	4 (2, 7)	5 (3, 7)	0.001*
Number of embryos	4 (2, 6)	2.5 (2, 4)	2 (1, 4)	2 (2, 4)	0.016*
Number of blastocyst	0 (0, 2)	0 (0, 1)	0 (0, 0)	0 (0, 1)	0.003*
Number of high-quality embryos	1 (0, 2)	1 (0, 2)	1 (0, 1)	1 (0, 2)	0.100

Kruskal-Wallis test

BMI, body mass index; AFC, antral follicle count; Gn days, Gonadotropin days; Gn dosage, Gonadotropin dosage; GnRH-ant days, Antagonist days; GnRH-ant dosage, Antagonist dosage;

Group P1MLH1: group with transiently premature luteinizing hormone surges, and P>1.5 ng/ml;

Group P1MLH0: group with no transiently premature luteinizing hormone surges, and P>1.5 ng/ml;

Group P0MLH1: group with transiently premature luteinizing hormone surges, and P ≤ 1.5 ng/ml;

Group P0MLH0: group with no transiently premature luteinizing hormone surges, and P ≤ 1.5 ng/ml.

### Pregnancy outcome between group PMLH and subgroup comparisons

The relationship between each group PMLH and pregnancy outcomes was analyzed and compared. As displayed in **Table 2**, BPR (P=0.034*), CPR (P=0.028*), and LBR (P=0.036*) were significantly different across the PMLH subgroups. In the pairwise comparison of group PMLH, the comparison between group P1MLH0 and group P0MLH0 showed a statistically significant difference (BPR: P=0.011*; CPR: P=0.005*; LBR: P=0.011*). The pairwise comparisons between other subgroups were no statistically significant.

**Table 2:** BPR, CPR and LBR between group PMLH.

	group PMLH				χ2	P	subgroup PMLH		χ2	P
	P1MLH1	P1MLH0	P0MLH1	P0MLH0			P1MLH0	P0MLH0		
BPR	12/15 (80%)	21/55 (38.18%)	49/86 (56.98%)	405/534 (75.84%)	8.652	0.034*	21/55 (38.18%)	405/534 (75.84%)	6.935	0.011*
CPR	10/17 (58.82%)	19/57 (33.33%)	48/87 (55.17%)	389/550 (70.73%)	9.082	0.028*	19/57 (33.33%)	389/550 (70.73%)	7.893	0.005*
LBR	8/19 (42.11%)	15/61 (24.59%)	43/92 (46.74%)	319/620 (51.45%)	6.653	0.036*	15/61 (24.59%)	319/620 (51.45%)	6.453	0.011*

χ2-test.

BPR, biochemical pregnancy rate; CPR, clinical pregnancy rate; LBR, live birth rate.

Group P1MLH1: group with transiently premature luteinizing hormone surges, and P>1.5 ng/ml;

Group P1MLH0: group with no transiently premature luteinizing hormone surges, and P>1.5 ng/ml;

Group P0MLH1: group with transiently premature luteinizing hormone surges, and P ≤ 1.5 ng/ml;

Group P0MLH0: group with no transiently premature luteinizing hormone surges, and P ≤ 1.5 ng/ml.

a. In the pairwise comparison of group PLH in BPR, CPR and LBR, the comparison between group P1MLH0 and group P0MLH0 was statistically significant (P=0.011*; P=0.005*; P=0.011*). The pairwise comparisons between other subgroups were no statistically significant.

### Correlation analysis

**Figure 1:**
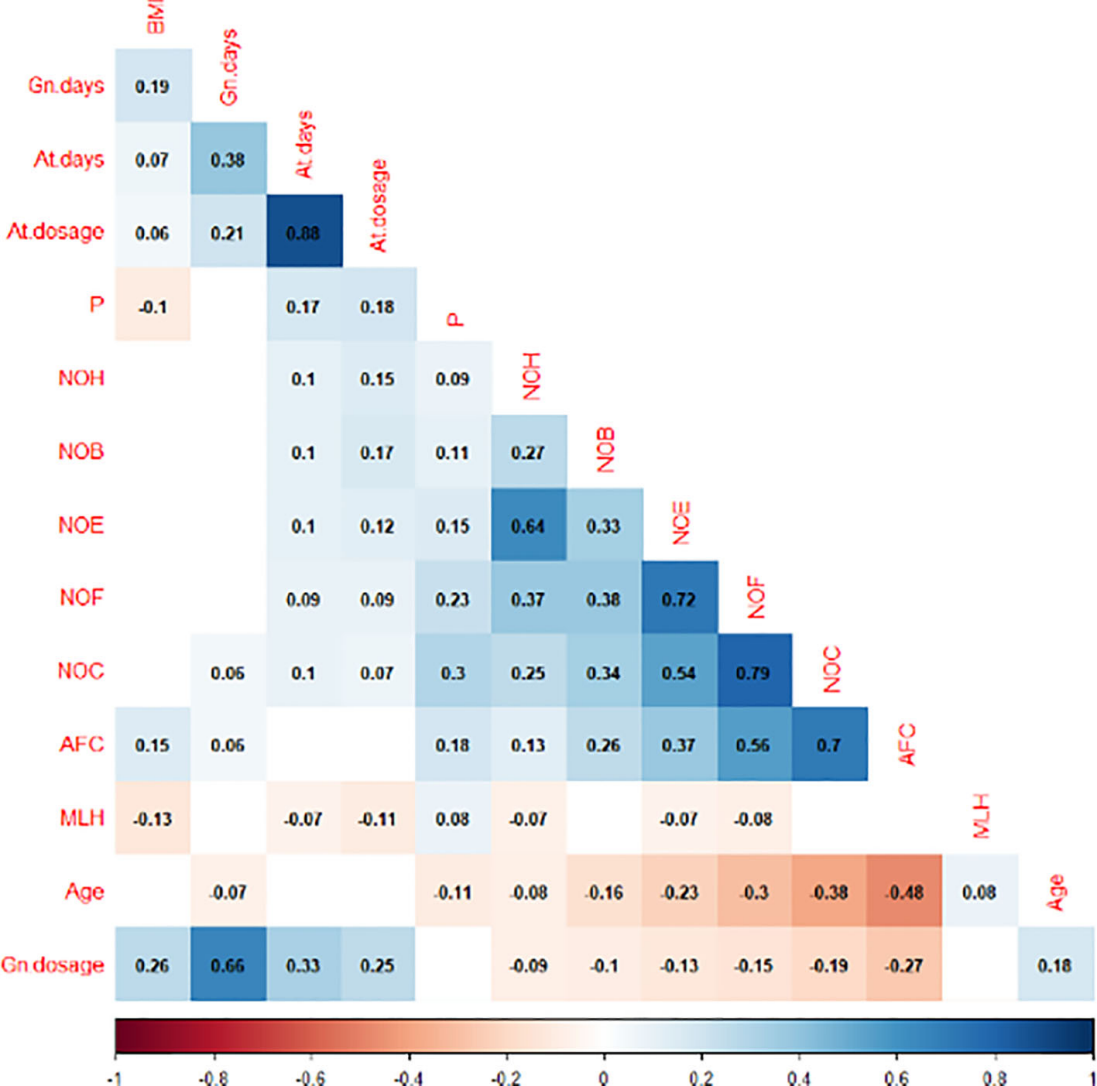
Correlation analysis between the maximum level of LH, P on the HCG day and other indicators.

**Figure 1** displays the correlation analysis results between the indicators. A significant negative association was found between the maximum LH level and BMI (R=-0.13, P=0.000*), GnRH-ant days (R=-0.07, P=0.018*), GnRH-ant dosage (R=-0.11, P=0.000*), and the outcome of COH, including number of fertilization (NOF) (R=-0.08, P=0.005*), number of embryos (NOE) (R=-0.07, P=0.023*), number of high-quality embryo (NOH) (R=-0.07, P=0.012*). In contrast, a significant positive association was observed between the maximum LH level and female age (R=0.08, P=0.004*). In addition, the same trend was observed between the maximum LH and P levels on the hCG day, indicating a positive correlation (R=0.08, P=0.005*). The P level exhibited a negative correlation with BMI (R=-0.1, P=0.000*) and age (R=-0.11, P=0.000*), while demonstrating a positive association with AFC (R=0.18, P=0.000*) and COH outcome, including number of oocytes (NOC) (R=0.3, P=0.000*), NOF (R=0.23, P=0.000*), NOE (R=0.15, P=0.000*), number of blastocyst (NOB) (R=0.11, P=0.000*), NOH (R=0.09, P=0.002*).

### Differences in correlation indicators

**Figure 2:**
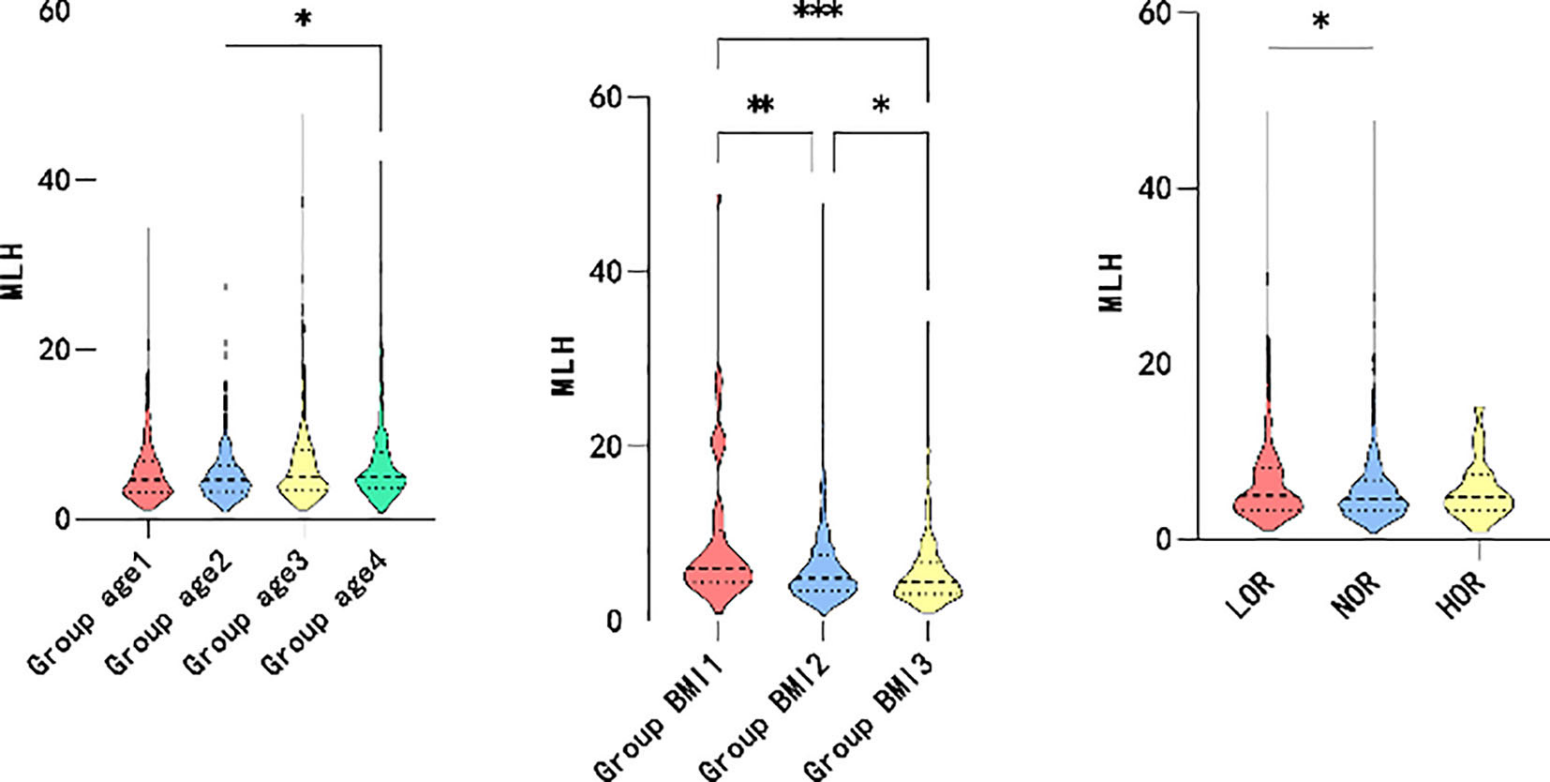
Maximum level of LH between group age, group BMI and different ovarian responses.

**Figure 3:**
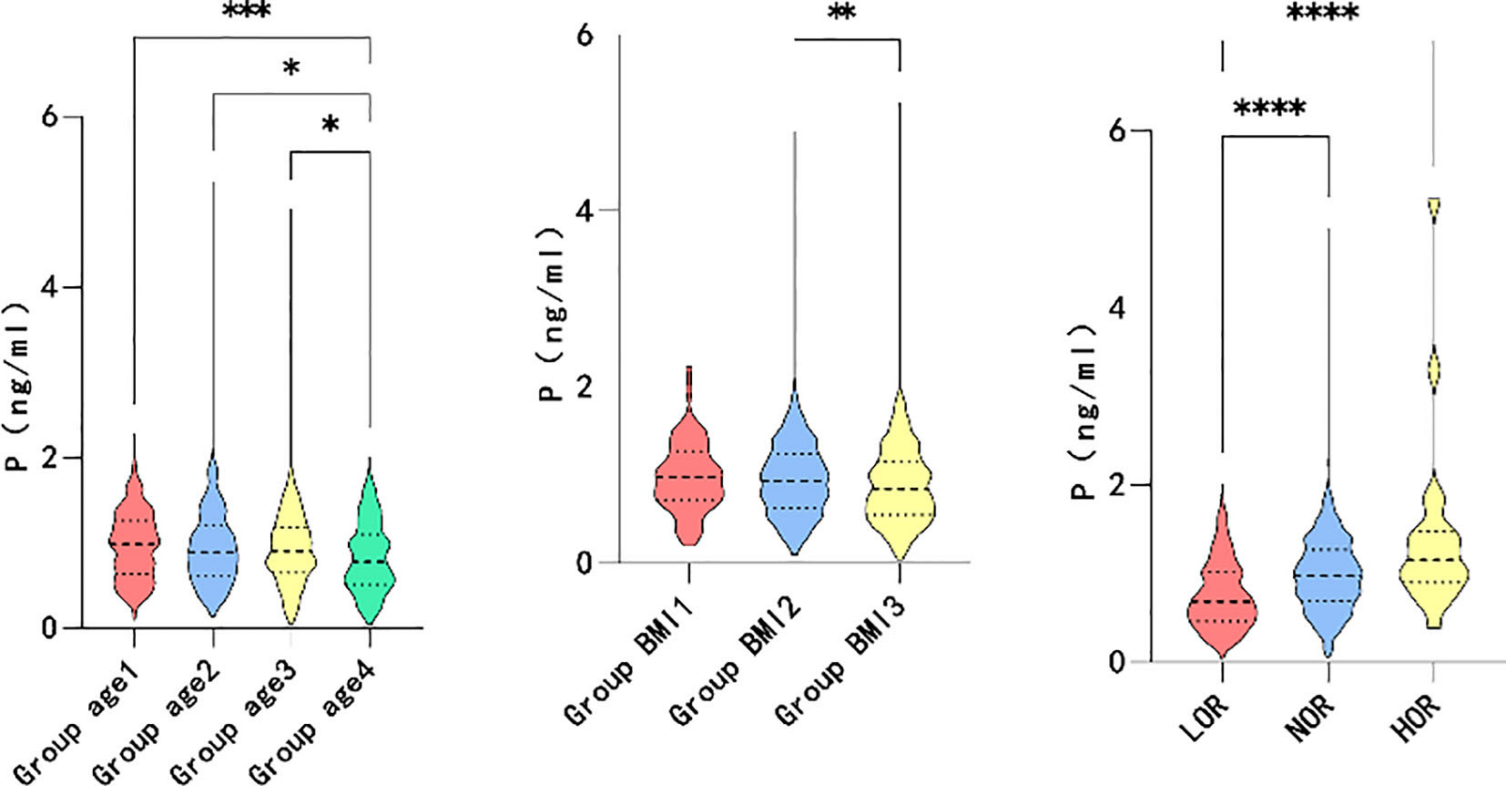
Level of P on the HCG day between group age, group BMI and different ovarian response.

Based on the correlation analysis results, the patients were further subgrouped by age and BMI. The participants were divided into quartiles of age ≤ 30, 30<age ≤ 34, 34<age ≤ 38, and 38<age. Moreover, the patients were subgrouped by BMI into BMI<18.5, 18.5 ≤BMI<24, and 24≤BMI based on guidelines for the prevention and management of overweight and obesity in the adult Chinese population (2004). High and low ovarian responses were defined as >18 oocytes retrieved and <6 oocytes retrieved, respectively. As shown in **Figure 2**, higher maximum LH levels were associated with older age (P=0.006*), lower BMI (P=0.000*), and decreased ovarian response (P=0.046*). However, elevated P levels on hCG day were associated with younger individuals (P=0.001*), participants with lower BMI (P=0.006*), and higher ovarian response (P=0.000*), as illustrated in **Figure 3**.

**Figure 4:**
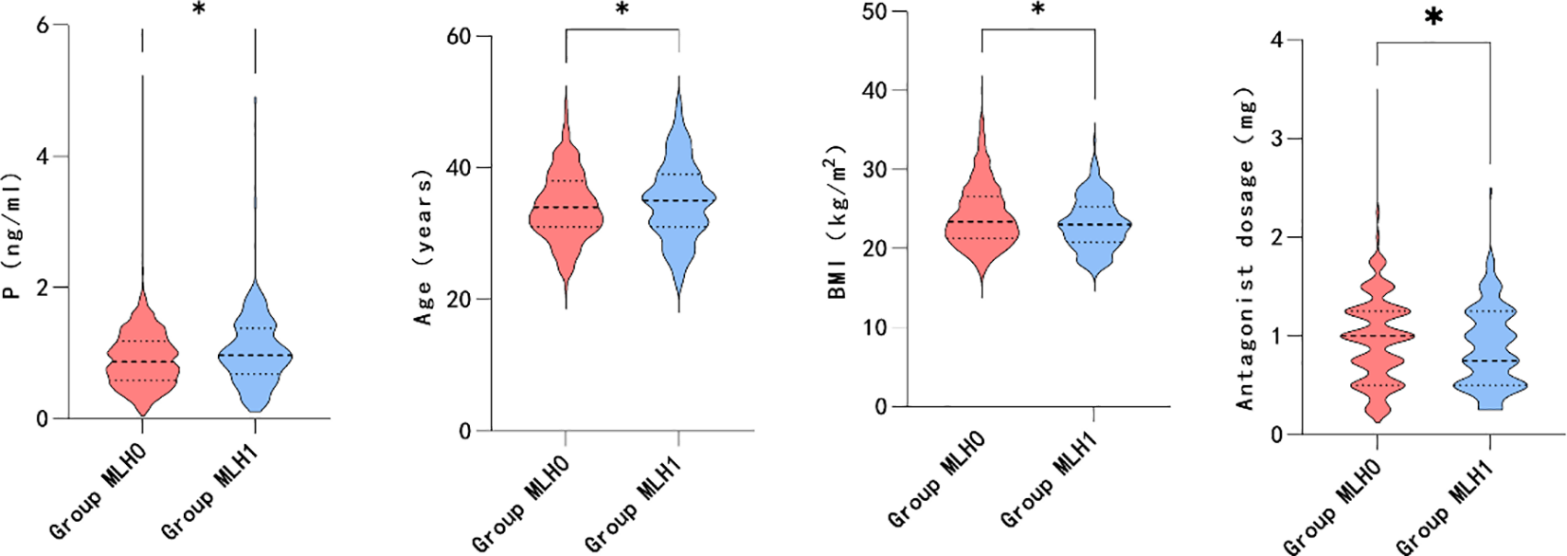
Level of P on the HCG day, female age, BMI and antagonist dosage between group MLH.

**Figure 5:**
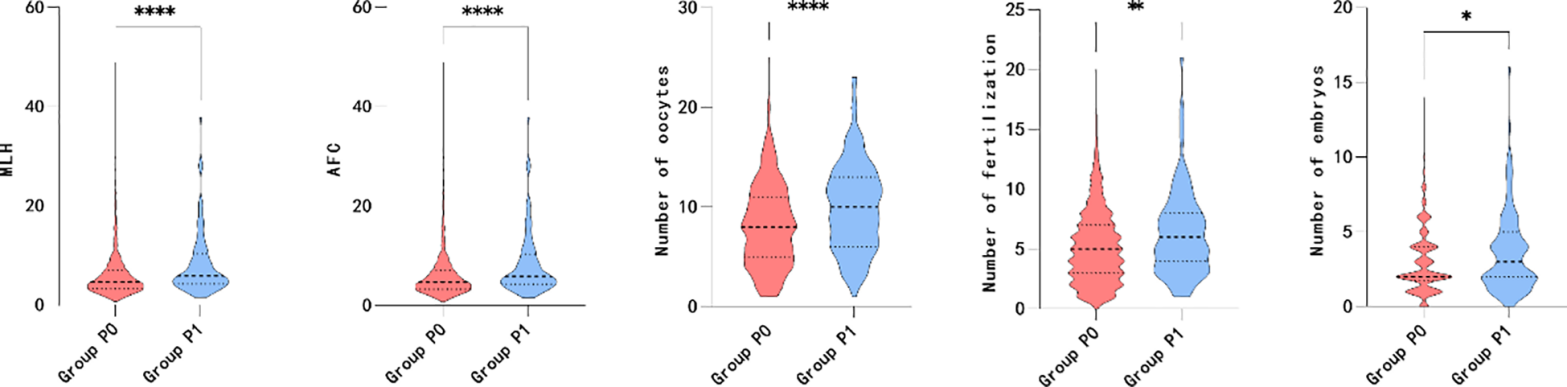
Maximum LH, AFC, number of oocytes, number of fertilization and number of embryos between group P.

Furthermore, the association between transient premature LH surge and the above subgroups was explored. The statistically significant indicators are shown in **Figures 4**, **5**. The patients with transiently premature LH surges had older age (P=0.045*), lower BMI (P=0.014*), lower GnRH-ant dosage (P=0.020*), and higher P levels (P=0.011*) on the hCG day. However, the increased P levels were mainly related to patients’ AFC (P=0.004*). Higher P levels were associated with a greater number of oocytes (P=0.000*), fertilization (P=0.002*), and number of embryos (P=0.046*).

### Effects of group PMLH on LBR

Binary logistic regression analysis revealed that group P1MLH0 (OR =0.432 [0.218–0.854]; P =0.016*) had opposite influences on the live birth rate compared with group P0MLH0, as indicated in **Table 3**. The same results were obtained in normal ovarian response (OR =0.49 [0.25–0.94]; P =0.03*), as indicated in **Table 4**.

**Table 3:** Binary logistic regression on LBR with the group PMLH.

			95% CI	
Dependent variable: LBR	P-Value	OR	Lower	Upper
Independent variables				
Age, years	.000*	.904	.874	.935
BMI, kg/m^2^	.100	1.034	.994	1.077
AFC	.731	1.003	.987	1.019
Gn dosage, IU	.085	1.000	1.000	1.000
Antagonist dosage, mg	.170	1.295	.895	1.872
Endometrial thickness, mm	.049*	1.069	1.000	1.143
Number of embryo transfers	.006*	1.790	1.186	2.702
Whether high-quality embryos	.010*	.662	.484	.906
Whether blastocysts	.201	.571	.242	1.348
Group PMLH	.055			
P1MLH1	.331	.596	.209	1.694
P1MLH0	.016*	.432	.218	.854
P0MLH1	.424	1.229	.741	2.039

The independent variables also included female age, BMI, AFC, Gn dosage, antagonist dosage, endometrial thickness, number of embryo transfers, whether high-quality embryos, whether blastocysts and group PMLH. We defined the group P0MLH0 as the last reference category.

LBR, live birth rate; BMI, body mass index; AFC, antral follicle count; Gn dosage, Gonadotropin dosage.

Group P1MLH1: group with transiently premature luteinizing hormone surges, and P>1.5 ng/ml;

Group P1MLH0: group with no transiently premature luteinizing hormone surges, and P>1.5 ng/ml;

Group P0MLH1: group with transiently premature luteinizing hormone surges, and P ≤ 1.5 ng/ml;

Group P0MLH0: group with no transiently premature luteinizing hormone surges, and P ≤ 1.5 ng/ml.

**Table 4:** Binary logistic regression on LBR with the group PMLH in NOR.

			95% CI	
Dependent variable: LBR	P-Value	OR	Lower	Upper
Independent variables				
Age, years	0.00*	0.91	0.88	0.94
BMI, kg/m2	0.33	1.02	0.98	1.07
AFC	0.90	1.00	0.98	1.02
Gn dosage, IU	0.13	1.00	1.00	1.00
Antagonist dosage, mg	0.05	1.12	1.00	1.26
Endometrial thickness, mm	0.50	1.01	0.98	1.04
Number of embryo transfers	0.01*	1.89	1.14	3.14
Whether high-quality embryos	0.01*	0.64	0.46	0.90
Whether blastocysts	0.14	0.52	0.21	1.24
Group PMLH	0.08			
P1MLH1	0.13	0.44	0.15	1.27
P1MLH0	0.03*	0.49	0.25	0.94
P0MLH1	0.92	1.03	0.61	1.74

The independent variables also included female age, BMI, AFC, Gn dosage, antagonist dosage, endometrial thickness, number of embryo transfers, whether high-quality embryos, whether blastocysts and group PMLH. We defined the group P0MLH0 as the last reference category.

LBR, live birth rate; BMI, body mass index; AFC, antral follicle count; Gn dosage, Gonadotropin dosage;

NOR, Normal ovarian response.

Group P1MLH1: group with transiently premature luteinizing hormone surges, and P>1.5 ng/ml;

Group P1MLH0: group with no transiently premature luteinizing hormone surges, and P>1.5 ng/ml;

Group P0MLH1: group with transiently premature luteinizing hormone surges, and P ≤ 1.5 ng/ml;

Group P0MLH0: group with no transiently premature luteinizing hormone surges, and P ≤ 1.5 ng/ml.

### Curve fitting between P and LBR with no transiently premature LH surges

**Figure 6:**
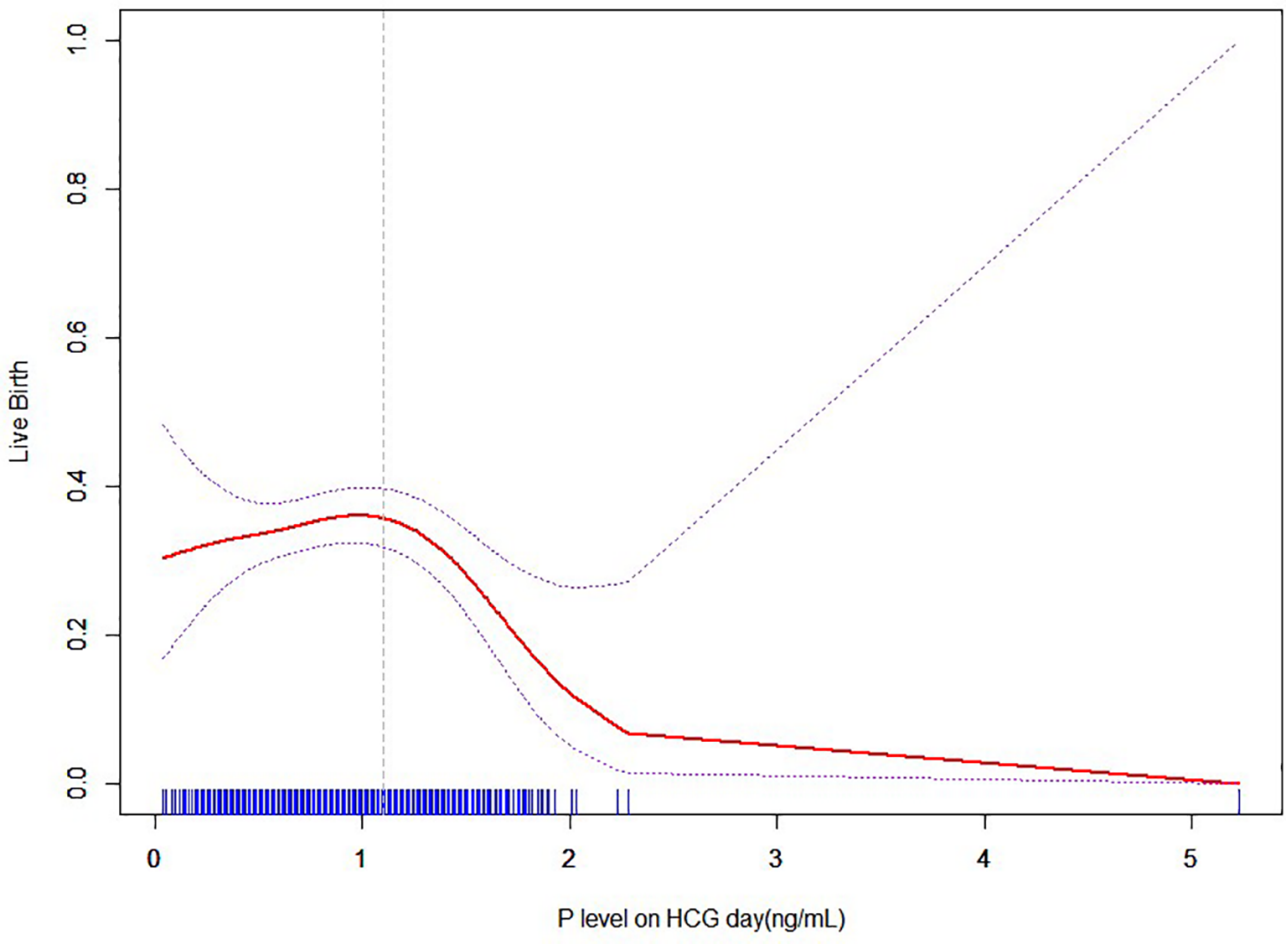
Nonlinear association between P level and live birth rate with no transiently premature LH surges.

**Figure 6** shows the fitted curves after adjustment for confounders between the P level on the hCG day and the live birth rate without transient premature LH surge. The results yielded a parabolic reverse-U curve as the P level increased. The curve initially increased before declining at the highest P level on the hCG day.

### Threshold effect analysis of P and LBR with no transient premature LH surge

The results of the threshold effect analysis of the P levels and LBR are presented in **Table 5**. The live birth rate showed a significant decrease when the P level was ≥1.1ng/ml.

**Table 5:** Threshold effect analysis between P level and live birth rate with no transiently premature LH surges.

			95% CI	
	P-Value	OR	Lower	Upper
P<1.10 (ng/ml)	0.222	1.454	0.798	2.651
P≥1.10 (ng/ml)	0.005*	0.25	0.09	0.66

piece-wise linear regression method

Adjusted for female age, BMI, AFC, Gn dosage, antagonist dosage, endometrial thickness, number of embryo transfers, whether high-quality embryos and whether blastocysts.

## Discussion

This study examined the maximum levels of luteinizing hormone (MLH) during controlled ovarian hyperstimulation (COH) and the P level on the hCG day. Patients with lower BMI, older age, lower ovarian response, and lower GnRH-ant dosage were more likely to develop LH surge. Although the LH surge could lead to a slight reduction in the number of embryos, there was no apparent correlation observed between the occurrence of LH surge and pregnancy outcomes with a fresh embryo transfer cycle. This finding aligns with part of the literature that suggests LH surge does not impact pregnancy outcomes.

The increase in P level on hCG day is also associated with the patient’s age, BMI, and ovarian response. Similar to LH levels, higher BMI was associated with a lower likelihood of progesterone elevation, which may be attributed to the increased Gn requirement for inducing rises in both progesterone and LH levels in patients with higher body weights. In contrast to the LH level, a higher incidence of elevated P levels was observed with younger age, higher number of AFC, and higher ovarian response. However, significantly poorer pregnancy outcomes were observed in patients with P levels exceeding 1.5ng/ml, which was consistent with previous literature. In our center, a P level on the day of hCG of more than 1.5 ng/ml was used as the threshold to decide whether to perform fresh embryo transfer. This threshold was also confirmed in the present study, demonstrating a clear decrease in the LBR.

Interestingly, while the LH surge was not associated with pregnancy outcomes, its occurrence was significantly associated with increased P levels on the hCG day. Moreover, it was worth considering that the presence of elevated progesterone levels may contribute to adverse pregnancy outcomes, while elevated progesterone levels were positively correlated with an increased number of oocytes retrieved, fertilized, and embryos. In addition, LH surge was more commonly observed in older patients and patients with diminished ovarian response. Conversely, a higher P level was typically found in younger patients and individuals with a higher ovarian response and higher AFC. Therefore, the patients were categorized into four groups based on the presence of the LH surge and P > 1.5ng/ml in order to investigate the impact of the LH surge and progesterone levels on live birth. Through pairwise combinations, no LH surge and P>1.5 ng/ml in the subgroup P1MLH0, resulting in a significantly lower live birth rate than group P0MLH0.

Furthermore, the patients were analyzed based on the PMLH grouping. The patients in group P1MLH0 had the highest GnRH-ant dosage and GnRH-ant days. The lowest pregnancy rate was observed in group P1MLH0, which might be related to the high dosage of GnRH-ant used, the elevated progesterone levels, and the occurrence of an occult LH surge. Poorer pregnancy outcomes were observed as the dosage of GnRH-ant increased, with exerting a detrimental impact on the receptivity of the endometrium and the development of embryos (36–38). However, the total GnRH-ant dosage was associated with the occurrence of LH surge. The appropriate range of GnRH-ant dosage for different patients lacks definitive studies at present, and further research is required.

When P ≤1.5ng/ml, the occurrence of LH surge (Group P0MLH1) was associated with advanced age, lower BMI, and lower GnRH-ant dosage. The numbers of embryos were the lowest in Group P0MLH1. However, after inter-group correction, no statistically significant differences were observed in IVF/ICSI outcomes and pregnancy outcomes among individuals experiencing an LH surge compared to those who did not. Conversely, when P>1.5 ng/ml, there was no notable distinction between the groups with or without LH surge.

In cases with an LH surge, elevated P levels did not have a detrimental impact on the pregnancy outcome of fresh embryo transfer, and positive correlation was observed between elevated P levels and improved outcomes in terms of numbers of oocytes, fertilization, embryos and blastocysts. This phenomenon could be attributed to enhanced embryo quality counteracting the potential negative impact of elevated progesterone levels on endometrial receptivity. In cases without LH surge, patients with P > 1.5 ng/ml (Group P1MLH0) had highest GnRH-ant dosage (P=0.049*), and lower oocyte numbers, fertilization numbers and embryos than group P1MLH1. Therefore, the poorest pregnancy outcome in the group P1MLH0 is more likely attributed to the receptivity of the endometrium or insufficient high-quality embryos to counterbalance adverse endometrial factors.

Our study may contribute to the establishment of an appropriate threshold for fresh embryo transfer without LH surge. Luteinizing hormone and progesterone levels were analyzed to investigate their effects on pregnancy outcomes. Our findings suggest that higher GnRH-ant dosages have a detrimental impact on both embryo quality and the pregnancy outcome after fresh embryo transfers. Additionally, when an LH surge occurs, increased levels of progesterone are associated with improved embryo outcomes; however, the pregnancy rate after fresh embryo transfer showed no significant improvement due to the negative effect of progesterone on endometrial receptivity. Conversely, in cases without LH surge, higher levels of progesterone led to lower live birth rates. Therefore, we recommend cryopreserving all embryos when progesterone levels exceeded or equaled1.10 ng/ml in order to mitigate the adverse effects of elevated progesterone and GnRH-ant dosage on live birth rates. Nevertheless, the retrospective nature of our study imposed certain limitations, necessitating further prospective studies to validate our findings.

## Data Availability

The original contributions presented in the study are included in the article/supplementary material. Further inquiries can be directed to the corresponding author.
